# Identification of therapeutically potential targets and their ligands for the treatment of OSCC

**DOI:** 10.3389/fonc.2022.910494

**Published:** 2022-09-20

**Authors:** Pratima Kumari, Sugandh Kumar, Madhusmita Sethy, Shyamlal Bhue, Bineet Kumar Mohanta, Anshuman Dixit

**Affiliations:** ^1^ Computational Biology and Bioinformatics Laboratory, Institute of Life Sciences, Bhubaneswar, India; ^2^ Regional Centre for Biotechnology (RCB), Faridabad, India

**Keywords:** chemotherapy, chemoresistance, prognosis, drug discovery, OSCC

## Abstract

Recent advancements in cancer biology have revealed molecular changes associated with carcinogenesis and chemotherapeutic exposure. The available information is being gainfully utilized to develop therapies targeting specific molecules involved in cancer cell growth, survival, and chemoresistance. Targeted therapies have dramatically increased overall survival (OS) in many cancers. Therefore, developing such targeted therapies against oral squamous cell carcinoma (OSCC) is anticipated to have significant clinical implications. In the current work, we have identified drug-specific sensitivity-related prognostic biomarkers (*BOP1*, *CCNA2*, *CKS2*, *PLAU*, and *SERPINE1*) using gene expression, Cox proportional hazards regression, and machine learning in OSCC. Dysregulation of these markers is significantly associated with OS in many cancers. Their elevated expression is related to cellular proliferation and aggressive malignancy in various cancers. Mechanistically, inhibition of these biomarkers should significantly reduce cellular proliferation and metastasis in OSCC and should result in better OS. It is pertinent to note that no effective small-molecule candidate has been identified against these biomarkers to date. Therefore, a comprehensive *in silico* drug design strategy assimilating homology modeling, extensive molecular dynamics (MD) simulation, and ensemble molecular docking has been applied to identify potential compounds against identified targets, and potential molecules have been identified. We hope that this study will help in deciphering potential genes having roles in chemoresistance and a significant impact on OS. It will also result in the identification of new targeted therapeutics against OSCC.

## Graphical Abstract

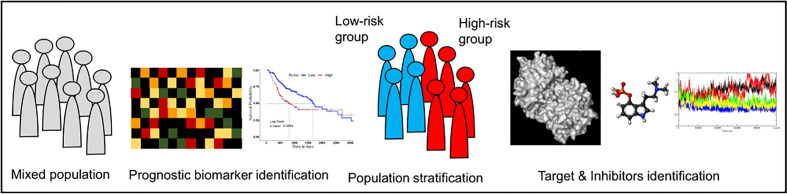

## Introduction

Oral squamous cell carcinoma (OSCC) constitutes a major subset of head and neck squamous cell carcinoma (HNSCC) and accounted for an estimated 0.37 million cases in the year 2020 (1). The high morbidity and mortality of OSCC pose a great challenge to its management (1, 2). The overall 5-year survival rate in OSCC is comparatively lower (~50%) than that in many other cancers (3, 4). For example, in India, 0.13 million new cases were detected, whereas 75,000 patients died in 2020 (1). Current treatment modalities include surgery, radiotherapy, chemotherapy, or their combinations. These are successful in patients with primary tumors, whereas patients with high-risk features (invasion/perineural invasion, metastasis, T3/T4 stage, or involvement of two or more lymph nodes) show less improvement. There is no clinical evidence to support the likely outcome in the case of high-risk oral cancer (5, 6).

Patient response to chemotherapy has been linked to tumor lineage and genetics. Changes in cellular gene expression in response to small-molecule treatments can provide insights into cellular processes governing the clinical outcome. Identifying the responsive features and mechanism of action is of immense value in cancer therapy and can be critical for the development of novel medicines. Furthermore, the heterogeneous response of patients to cancer therapies and the frequent development of drug resistance highlight the importance of a therapeutic response (7, 8). However, an accurate prediction of a therapeutic response and the identification of new anticancer drugs have remained a challenging task.

In recent years, the increasing understanding of genomics and the advent of next-generation sequencing (NGS) with advancements in bioinformatics approaches have made it possible to identify potential molecular targets for the betterment of chemotherapy. Numerous cancer studies with the help of NGS were able to identify novel and rare somatic mutations in more efficient and accurate ways. In a variety of cancers such as bladder cancer, renal cell carcinoma, small-cell lung cancer, prostate cancer, acute myelogenous leukemia, and chronic lymphocytic leukemia, researchers were able to accurately identify genetic alterations. On the other hand, together with NGS, the bioinformatics approach was successful in exploiting the heterogeneous nature of cancer to develop cancer diagnostic, prognostic, and predictive markers (9). Gene-based approaches are being used for the development of new therapeutic agents. The targeted approaches have been proven highly useful in the development of therapies for many cancers (10).

Doxorubicin is known to inhibit the topoisomerase-II (TOP2) activity in eliciting its antineoplastic effect. It is one of the most effective anticancer drugs widely used in the treatment of several cancers including breast cancer, lung cancer, gastric cancer, ovarian cancer, thyroid cancer, non-Hodgkin’s and Hodgkin’s lymphoma, multiple myeloma, sarcoma, and pediatric cancer (11). High toxicity and early resistant phenotype have limited the use of doxorubicin (12). Thus, it is imperative to study the molecular changes associated with doxorubicin resistance. Therefore, in the current work, we aimed to explore gene expression changes in the response to doxorubicin treatment in OSCC using various datasets and the ways to reduce the chances of emergence of such resistance by molecularly targeted therapies. We have also identified potential ligands that may increase the effect of doxorubicin and/or can delay the progression of drug-related resistant phenotypes or sensitize oral cancer cells to chemotherapy.

## Materials and methods

### Data collection and differential gene expression analysis

In the present study, data were collected from two sources: The Cancer Genome Atlas (TCGA) (https://portal.gdc.cancer.gov/) and Gene Expression Omnibus (GEO) (https://www.ncbi.nlm.nih.gov/geo/). Since we wanted to analyze OSCC data, we further filtered out the OSCC samples according to the International Classification of Diseases (ICD) code. The ICD classifies diseases based on the site of disease occurrence (**Supplementary Table S1**). Finally, 319 OSCC and 44 normal adjacent tissue (NAT) samples were obtained. Additionally, two more datasets were obtained from GEO (13, 14): 1) mRNA expression profiling data of 27 OSCC patients (GEO ID-GSE23558) and 2) mRNA expression profiling data of the doxorubicin-treated SCC25 cell line (GSE58074). Two replicates of each (mock and treatment) were taken for expression analysis. The clinical details of TCGA patients are given in **Supplementary Table S2**.

The gene expression analysis was performed using R studio (http://www.rstudio.com/) version 3.4.4 using the limma-voom library for TCGA samples, whereas GEO samples were analyzed using GEO2R. Genes with |log2FC ≥1| and p-value<0.05 were considered significantly differentially expressed genes (DEGs). The ggplot2, complex heatmap, and circular library in R were used for volcano plot and heatmap. Common DEGs in all of the three datasets were considered genes of interest for further analysis.

### Functional enrichment analysis

Gene Ontology (GO) and pathway enrichment of common genes present in all of the datasets were used to identify enriched biological events as a result of doxorubicin perturbation. We have used Reactome (https://reactome.org) (15, 16) and GO (http://geneontology.org/) (17, 18) online databases for enrichment analysis of the gene sets. Enriched biological pathways and GO terms were considered significant if p-value ≤0.05.

### Cox proportional hazards regression

Cox proportional hazards regression (Coxph) is a semiparametric model used to predict the outcome of disease based on one or more predictors in survival time (time-to-event) through the hazard ratio (HR) function. It assumes that the effects of predictor variables have an additive effect on the hazard, i.e.,


(1) $$h\left(t\right)\;=\;h_{0}\left(t\right)\;\exp\;\left(\beta_{1}x_{1}\;+\cdots \cdots \;+\;\beta_{n}x_{n}\right)$$

where *h*(*t*) is the hazard at time *t* for a subject with a set of predictors *x*_1_…*x*_n_, *h*_0_(*t*) is the baseline hazard function, and β_1_,.,β_n_ are the model coefficients describing the effect of the predictors on the overall hazard. HR is used for the interpretation of the Cox model. The HR examination shows selective factors that influence the rate of an event happening (e.g., death) at a particular point in time. An HR above 1 indicates a predictor that is positively associated with the event probability (death) and thus negatively associated with the length of survival, indicating worse prognosis; a negative HR indicates a protective effect of the predictors with which it is associated; while an HR equal to 1 means no effect. The DEGs were subjected to a Cox regression analysis using the “survival” package in R (https://github.com/therneau/survival).

### Identification of biomarker signature and validation of prognosis-related genes

Machine learning (ML) is becoming popular in cancer biology in identifying prognostic, diagnostic, and therapeutic biomarkers. Therefore, we implemented two commonly used machine learning algorithms, *viz.*, random forest (RF) and partial least square (PLS) regression method, to identify prognostic targets. RF is a frequently used algorithm for the identification of prognostic biomarkers in many diseases including cancer (19, 20).

### Survival analysis

To evaluate the reliability of the predicted prognosis signature, Kaplan–Meier (K-M) plots were generated to estimate the survival of the patients based on their median mRNA expression. An mRNA expression above the median value was considered high, whereas an expression below the median was counted as low expression. The R package “survival” was used to plot the patient’s overall survival (OS), and significance was calculated based on the log-rank p-value. To further reflect the sensitivity and specificity of signature mRNAs, we employed a time-dependent receiver operating characteristic (ROC) curve analysis *via* the R package “survivalROC.”

### Risk assessment and validation of the risk model

The risk score is an additive model of the mRNA expression level multiplied by their Cox regression coefficient. The risk score was calculated as follows:


(2) Risk score = Coefficients of signature Gene A × mRNA expression of A + ………. + Coefficients of signature Gene N × mRNA expression of N

The median risk score was used to divide the patients into high- and low-risk groups.

### Use of the protein–protein interaction network to understand the potential signature biomarker

Next, we constructed the protein–protein interaction (PPI) network to understand the importance of key targets in the human interactome. The Search Tool for the Retrieval of Interacting Genes/Proteins (STRING) database (https://string-db.org/) was used to create a PPI network of identified genes with a cutoff score of >0.4, which was equivalent to medium confidence. Subsequently, a cluster analysis using MCODE (21) was done to decipher the modules in the created network. To understand the functional significance of the modules, pathway analysis was done using Reactome (www.reactome.org).

### Identification of candidate small-molecule drugs

#### Protein structures

The X-ray crystal structures of SERPINE1 [Protein Data Bank (PDB): 4AQH], CCNA2 (PDB: 1H1R], PLAU (PDB: 1OWE), and CKS2 (PDB: 5HQ0) were used in the current studies. The three-dimensional (3D) structure of BOP1 is not available in the PDB.

#### Homology modeling

The 3D structure of BOP1 was modeled by a homology modeling approach using modeler v9.20. Delta-blast was used to identify suitable templates for modeling against the PDB database. The template was selected based on the expected value (E-value), bit score, and percentage of query coverage and identity. The carboxy-terminal domain of Erb1 of *Escherichia coli* (PDB-ID: 4U7A chain A, identity 40.1%, coverage 83%) was finally selected as the template to model the structure of BOP1. Twenty models were generated for each protein, and the final model was selected based on the lowest molpdf score.

### System setup and molecular dynamics simulation

In the current study, the preliminary topology and coordinates for all proteins were generated in VMD v1.93, whereas the simulations were run in NAMD v2.14. All protein structures (BOP1, PLAU, SERPINE1, CKS2, and CCNA2 after stripping cocrystallized ligands) were prepared and solvated in a rectangular water box (TIP3P water model) with a buffering distance of 10 Å. Ions were added to ensure the electroneutrality of the solvated system. The SETTLE algorithm was used for the water molecules. The associated system topology and coordinates were generated by applying charmm34 force field parameters for molecular dynamics (MD) simulation. Prior to the simulation, the system was properly minimized with a stepwise minimization protocol. Firstly, the water molecules and ions were minimized that was then followed by hydrogen atoms and the side chains of the complex. The side chains were minimized for 40,000 steps, whereas the backbone atoms and the bond lengths of hydrogen atoms were kept fixed. Thereafter, all of the atoms were allowed to relax freely, and the whole system was energy-minimized for 40,000 steps with nominal restraints on Cα atoms (10 kcal/mol) to prevent any abrupt change in the structure. Subsequently, an equilibration protocol was followed where the system was heated gradually from 0 to 310 K in steps of 30 K with a canonical ensemble [constant volume, constant temperature (NVT)]. At each step, a 20-picosecond (ps) simulation was run to allow the system to adjust to the temperature. Once the system attained 310 K, an isobaric and isothermic ensemble [constant pressure, constant temperature (NPT)] was applied for a period of 100 ps with a constant pressure of 1.0 bar using Langevin dynamics. Finally, the applied restraints on Cα atoms were removed, and the system was equilibrated for 1 ns at 310 K using the Langevin piston coupling algorithm. During the whole simulation, the Particle Mesh Ewald (PME) algorithm was used to calculate the long-range electrostatic interactions with fixed periodic boundary conditions. The covalent bond interactions involving hydrogen were constrained using the SHAKE algorithm. Once the system was simulated with a constant 310 K temperature and 1.0 bar pressure, then the production run was done for a time period of 100 ns. The analyses of the MD trajectories were performed to analyze the structure and dynamic behavior of all proteins during MD. The trajectories were analyzed for root mean square deviation (RMSD) and radius of gyration (Rg); these analyses were performed using VMD and in-house Perl and Tcl scripts. Five additional equidistant frames were generated from each trajectory for ensemble docking studies.

To study the stability of the protein–ligand complex, another simulation was run for 300 ns with selected ligands docked in the protein targets. The ligand parameters were generated using the Antechamber module of AMBER12 molecular simulation package (www.ambermd.org). The complex was solvated in a box of water with 10 Å buffering distance. AMBER parameters and coordinate files were generated using the tleap module of AMBER12. Equilibration and simulation were done using NAMD v2.14 as described above. The trajectories were analyzed for RMSD, hydrogen bonds, and salt bridges to assess the stability and strength of the interactions between the ligand and the protein.

### Molecular docking

**Figure 1:**
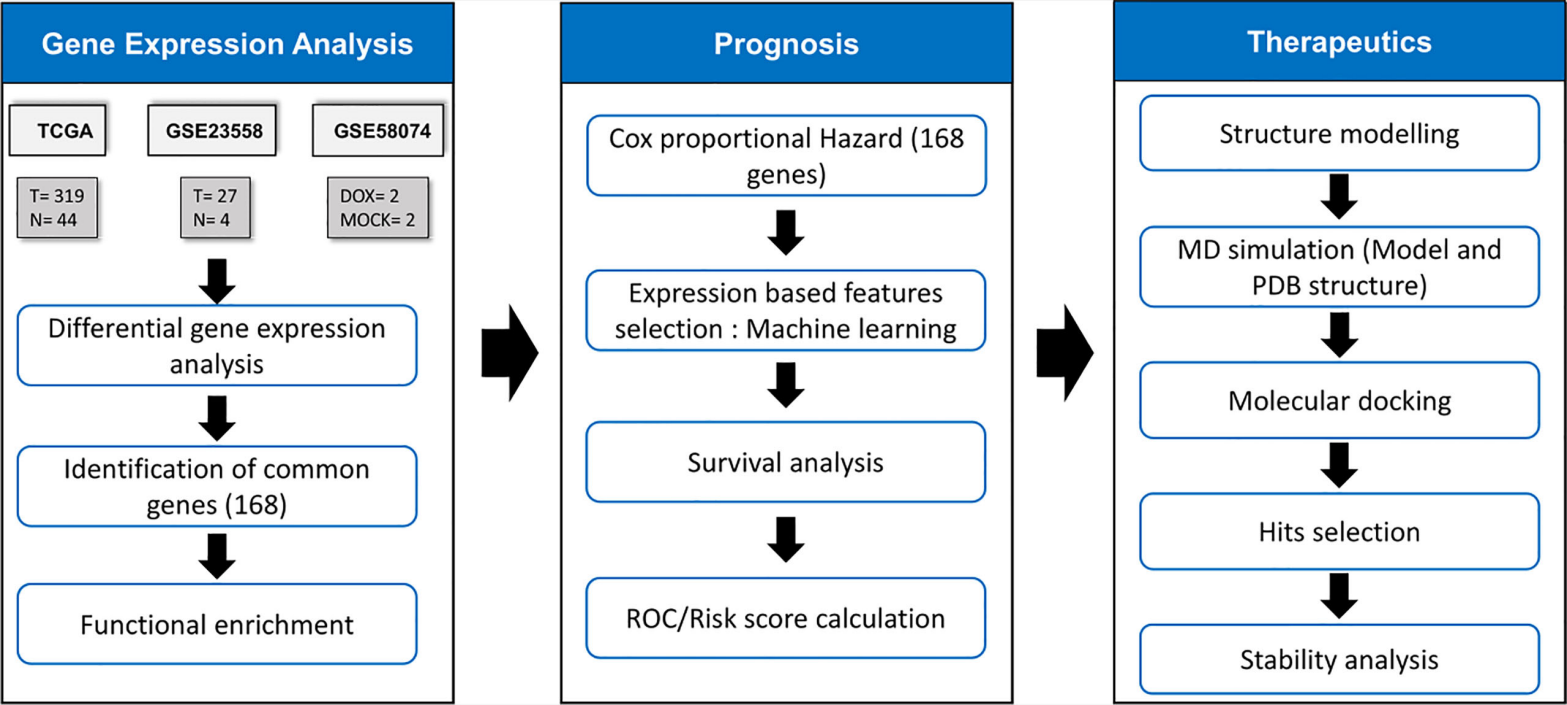
Methodology for identification of drug response related signature and identification of their inhibitors. The TCGA samples had 319 OSCC & 44 normal samples. GSE23558 had 27 OSCC patients’ data. GSE58074 examined the effect of doxorubicin on SCC25 cell lines to check for molecular markers underlying doxorubicin response..

The frames obtained from the MD simulation for the selected proteins were prepared using the protein preparation wizard module of Schrödinger molecular modeling software v9.3. OPLS-2005 force field was used for energy minimization, whereas Schrödinger’s LigPrep module was employed to generate 3D conformers and energy minimization of the potential small-molecule drugs from the US Food and Drug Administration (US FDA) library. A maximum of 32 conformers were generated per ligand. Before docking, the grid box (active site) was defined as residues within 5 Å of cocrystallized ligands. The active site of the modeled proteins or proteins without cocrystallized ligands was predicted through the sitemap algorithm as implied in Schrödinger. Docking was performed using standard precision (SP) mode with flexible ligand sampling in Schrödinger’s Glide module. The average docking score was calculated based on the glide score in the five frames. The flowchart of methodology is given in **Figure 1**.

## Results

### Identification of differentially expressed genes

**Figure 2:**
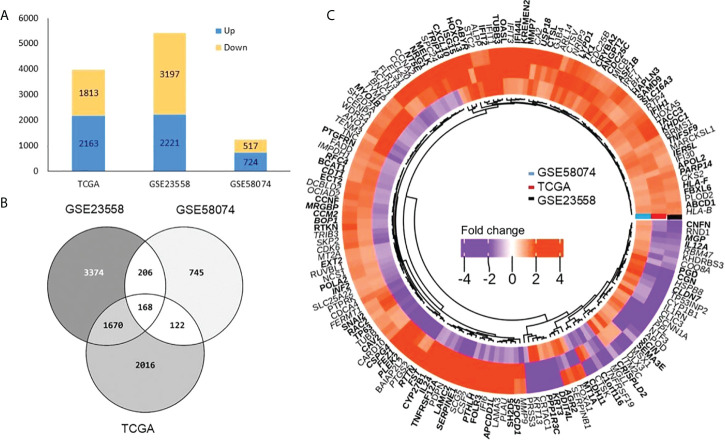
Differentially expressed genes **(A)** Bar graph representing total DEGs both up (blue) and down (yellow) in three datasets. **(B)** Venn diagram showing number of DEGs (common and unique) among TCGA, GSE23558 and GSE58074. A total of 168 common DEGs were obtained. **(C)** Heatmap shows DEGs related to doxorubicin response (GSE58074) and non-treated samples in OSCC from TCGA and GSE23558. Three types of distinct expression pattern are discernible (1) 52 genes overexpressed (orange) in all the three data sets, (2) 76 genes underexpressed in GSE58074 (purple) while overexpressed in TCGA (orange) and GSE23558 (orange), and (3) 26 genes underexpressed in TCGA (purple) and GSE23558 (purple) while overexpressed in GSE58074 (orange).

We identified 3,976, 5,418, and 1,241 DEGs in TCGA, GSE23558, and GSE58074 datasets, respectively. In TCGA samples, 2,163 genes were overexpressed and 1,813 genes were underexpressed, whereas 2,221 and 3,197 genes were found overexpressed and underexpressed, respectively, in GSE23558. In GSE58074, which contained doxorubicin-treated cell line data, 724 genes were overexpressed and 517 genes were underexpressed (**Figure 2A** and **Supplementary Table S3**). Among the three datasets, 168 common DEGs were identified. These were considered for further analysis (**Figure 2B**). DEGs are represented in a volcano plot with log_2_FC and -log_10_ p-value (**Supplementary Figure S1**). The heatmap represents the expression of common DEGs from the three datasets, with 134, 132, and 85 overexpressed genes and 34, 36, and 83 underexpressed genes in TCGA, GSE23558, and GSE58074, respectively (**Figure 2C** and **Supplementary Table S3**). From the heatmap, it is clearly visible that genes that are overexpressed in cancer prior to any treatment get downregulated after treatment and *vice versa*, whereas some of the genes present do not respond to the treatment, as there are no changes in their expression patterns.

### Functional enrichment analysis

**Figure 3:**
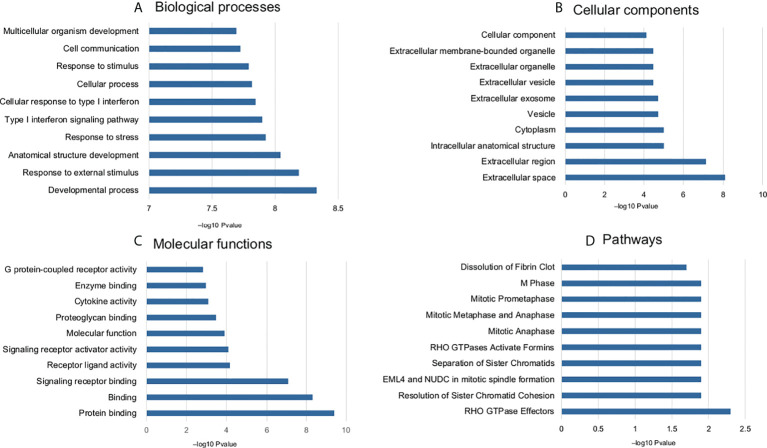
Enriched GO terms and pathways (top 10) common DEGs associated with doxorubicin response. **(A)** biological process, **(B)** cellular components, **(C)** molecular function, and **(D)** pathways.

The common DEGs (n = 168) were studied for functional enrichment to investigate their involvement in various cellular processes. The top 10 enriched GO functions and pathways are shown in **Figure 3**. Biological process (BP) terms related to immune response, biological stress, cell proliferation, and survival were found to be significantly enriched. Enriched cellular component (CC) terms include extracellular related components, protein complex, membrane-bound components, and cellular vesicles. Molecular function (MF) enriched terms included various signaling pathways involved in ligand–receptor complex, enzymatic functions, G protein–related function, kinases, and other protein-binding functions. Pathway enrichment shows that most of the signaling related to cell cycle and its regulation such as G2/M transition, nucleotide salvage pathways, and DNA damage bypass. The detailed pathway and GO term information is provided in **Supplementary Table S4** and **Supplementary Figure S2**.

### Exploration of prognostic biomarkers

**Figure 4:**
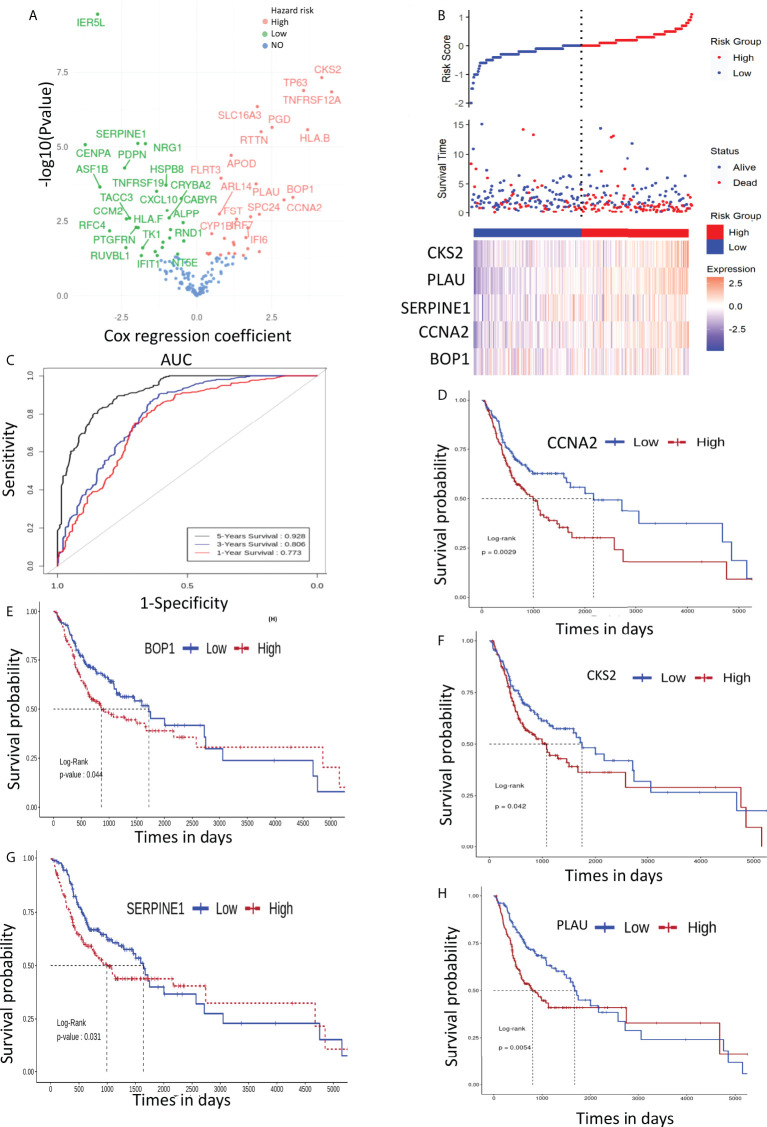
**(A)** Volcano plot of cox regression showing hazard ratio (HR) for 168 genes. **(B)** Patient stratification according to risk score to predict the survival time of patients with high- and low-expression level of prognostic genes. **(C)** ROC depicting the effect of selected genes on overall survival (1, 3, and 5 years). **(D–H)** depict the effect of signature gene expression on overall survival of OSCC patients..

The prognostic significance of the selected DEGs (168 genes) was assessed through univariate Coxph regression analysis. A total of 59 genes were found to be significantly (p-value<0.05) associated with OS (**Figure 4A** and **Supplementary Table S3**). As indicated earlier, the higher values of HR (HR >1) indicate worse prognosis, whereas negative HR values (<-1) show favorable prognosis. Next, machine learning algorithms, *viz.*, RF and PLS regression, were employed to identify the potential predictive prognostic signature genes. We selected the top predictive genes based on the robustness of the prediction. A cutoff percentile score of ≥80 was used; nine genes were selected from RF, whereas seven genes were predicted by PLS (a total of 16) (**Supplementary Table S5**). Then, we performed survival analysis to identify the potential prognostic markers. We found that five genes, *viz.*, *BOP1*, *PLAU*, *SERPINE1*, *CCNA2*, and *CKS2*, were significantly related to the survival of the patients. The risk assessment score was calculated with the help of Cox regression analysis that resulted in the following equation (Eq. 2) and was used to determine the risk score (high and low):


(2) $$\text{Risk score }=\;\left(3.207785\;\times \;{\text{expression}}_{BOP1}\right)\;+\;\left(1.986074\;\times \;{\text{expression}}_{PLAU}\right)\;-\;\left(1.962924\;\times \;{\text{expression}}_{SERPINE1}\right)\;+\;\left(2.913185\;\times \;{\text{expression}}_{CCNA2}\right)\;+\;\left(4.164371\;\times \;{\text{expression}}_{CKS2}\right)$$

Importantly, low-risk patients have a low expression of these mRNAs as compared with those of high-risk patients (**Figure 4B**). It can be seen clearly in **Figures 4D–H** that the low–risk score group had better OS; therefore, the expression of the identified mRNAs has significant impact on the OS of the patients. To further assess the accuracy of the prognostic model, we constructed an ROC curve to assess the impact of the expression of these genes on patients’ OS for 1, 3, and 5 years. The ROC curve is shown in **Figure 4C**, and the area under the curve (AUC) was 0.773, 0.806, and 0.928 for 1, 3, and 5 years, respectively.

### Assessment of the Five Prognostic Signature Genes Across Cancers

**Figure 5:**
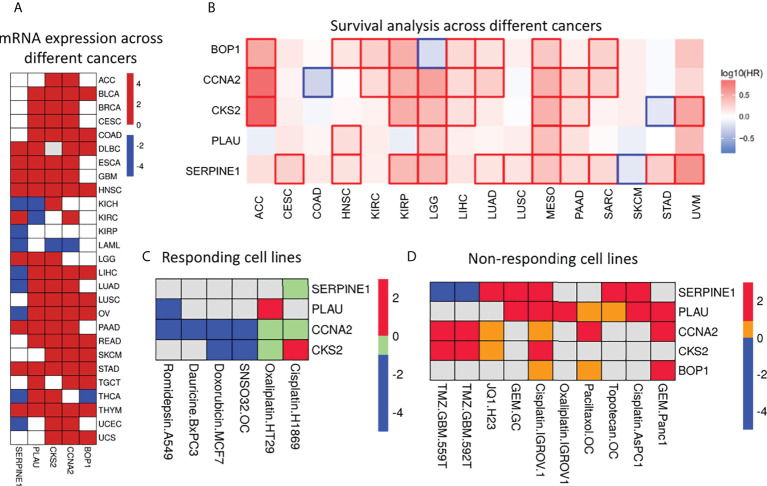
Expression & significance of selected prognostic genes. **(A)** Differential gene expression analysis across different cancers. Red, blue and white squares depict over, under and insignificant expression respectively. It is clear that these genes are upregulated in most of the cancers. **(B)** Survival map: Red and blue squares indicate poor survival due to over and under expression respectively. The figure clearly indicates the expression of these genes have significant effect on survival of the patients across cancers. **(C)** Heatmap represents expression level of these genes in drug sensitive cells. These genes were found to be significantly differentially expressed (|log_2_Fc| > 1 and pvalue < 0.05). The down- (blue) and up- (red) regulated genes in response to drug; green square indicates down-regulated genes with significant pvalue but |log_2_Fc| < 1. Grey square indicated no differential expression. **(D)** The expression of selected genes in drug resistant cells. Red and blue squares indicate significant (|log_2_Fc| > 1 and pvalue < 0.05) up- and down-regulated genes; yellow square indicates up-regulated genes with significant pvalue (|log_2_Fc| < 1); grey square indicated no differential expression of the genes.

The Cox regression and Machine learning (ML) analysis showed that overexpression of the identified genes is related to poor prognosis. Furthermore, we investigated the expression of these genes across different cancers and OS of patients to assess their clinical importance. For this, we have used GEPIA2 database (22). GEPIA2 retrieves data from TCGA and the Genotype-Tissue Expression (GTEx) portal. The expression analysis showed that these five genes were significantly upregulated (|log2FC >1|, p-value<0.05) in most of the tumor tissues as compared with those in normal tissues (**Figure 5A** and **Supplementary Table S6**). Survival analysis across various cancers showed that a high expression of these five genes is related to poor survival in most of the cancers (**Figure 5B**). Moreover, we also checked the expression of these genes in response to drug treatment. For this, we analyzed the expression array data available in the NCBI-GEO dataset (**Supplementary Table S7**). Two types of analysis were done: 1) expression analysis in drug-sensitive cells and 2) expression analysis in drug-resistant cells (**Figures 5C, D** **)**. We found that the mRNA expression level decreased for most of the genes upon drug treatment. For example, *CCNA2* mRNA levels were found to be decreased significantly with fold change -1.07, -4.33, -1.06, and -4.17 when treated with romidepsin, SNAO32, dauricine, and doxorubicin in A549, primary ovarian cancer, BxPC3, and MCF7 cell lines, respectively. Similarly, *PLAU*, *CKS2*, and *SERPINE1* mRNA levels were also found to be decreased in response to various drugs, whereas in the case of the drug-resistant cell line, an increase in mRNA expression levels of *PLAU*, *CKS2*, *CCNA2*, *BOP1*, and *SERPINE1* was observed. For example, PLAU showed increased mRNA expression with fold changes of 2.14, 1.8, 1.5, 1.4, and 1.2 in Panc1 gemcitabine-, MKN28 gemcitabine-, AsPc1 cisplatin-, IGROV1 cisplatin-, and IGROV1 oxaliplatin-resistant cell lines, respectively. Additionally, we analyzed the presence of genetic alterations (e.g., amplification, mutation, deletion, structural variant) in these five genes using CBioPortal (https://www.cbioportal.org/), which is an online consortium for cancer genomics. We investigated the TCGA Pan-Cancer Atlas dataset for the genomic alteration study (23, 24). It clearly showed that amplification is the most common alteration observed in *BOP1*, *PLAU*, *SERPINE1*, and *CKS2* across the cancers, whereas in *CCNA2*, mutation and deep deletion are prevalent (**Supplementary Figure S3**).

### Protein–protein interaction network

**Figure 6:**
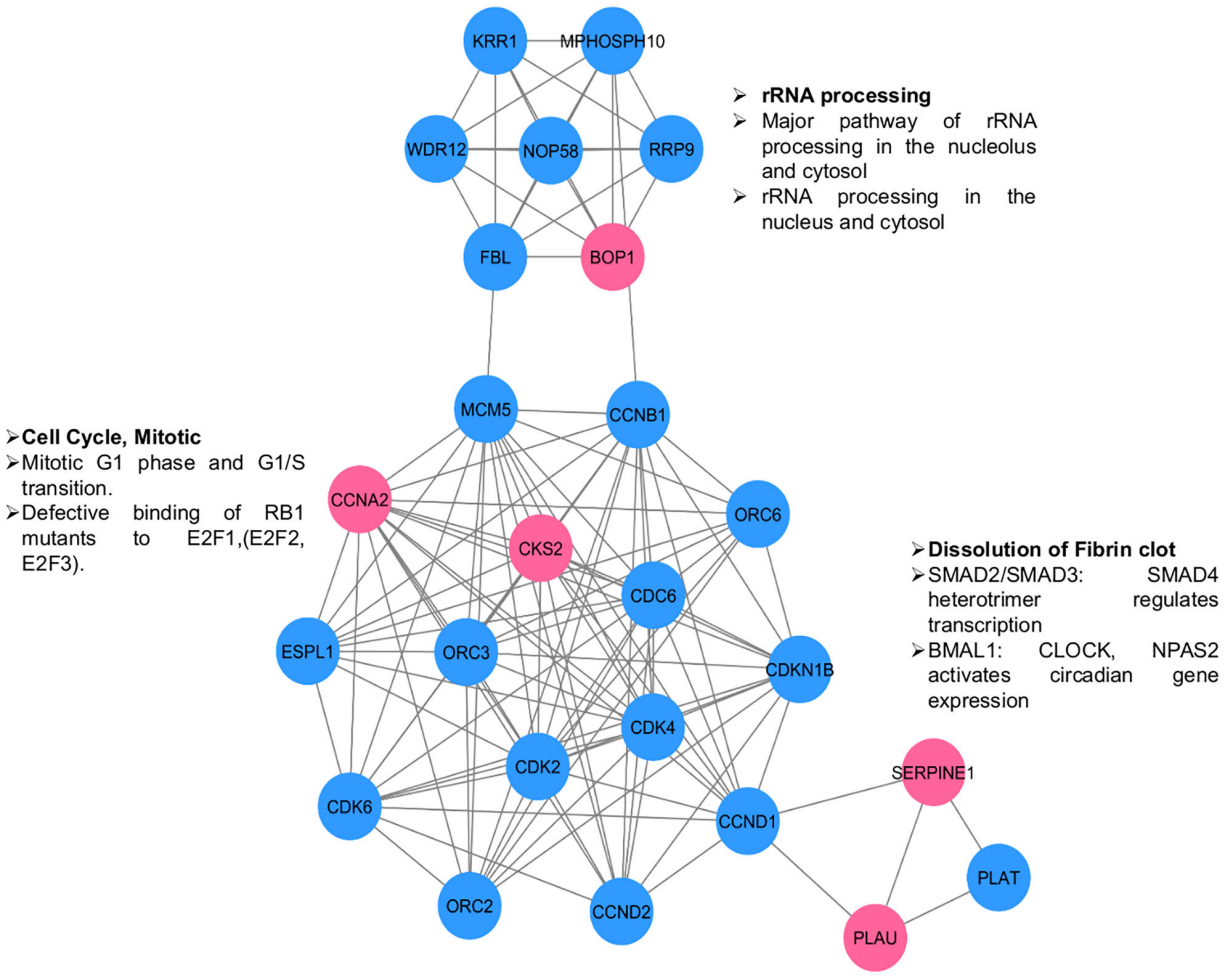
PPI network for selected genes (pink circles) to understand their interactions & significance. Other interacting proteins are depicted in blue circles. Three clusters are visible. The enriched functions are shown along the clusters.

The PPIs are at the heart of various molecular mechanisms. Therefore, a PPI network was constructed to understand the interactions of selected proteins with other proteins in the human interactome for better understanding of their regulatory roles (**Figure 6**). We identified three distinct clusters in the network; the proteins in those clusters are involved in 1) the regulation of rRNA processing; 2) the regulation of the cell cycle; and 3) angiogenesis, growth factors, and transcription factors such as Suppressor of Mothers against Decapentaplegic (SMAD) SMAD2/SMAD3. The overall network analysis indicates that these genes are connected to many important genes, and targeting them will affect cellular processes playing critical roles in the pathogenesis of OSCC.

### Molecular dynamics simulations

The MD analysis was performed to assess the flexibility of the binding site that is not discernible through PDB structures. The generated structural ensembles were used for the identification of small-molecule binders. MD simulations were done for 100 ns on each protein (total 500 ns), and the stability of the simulation was evaluated using RMSD. The RMSD values reveal the structural changes that occurred during the MD. The RMSD plots for all proteins indicated that each system was stabilized quickly and then remained stable throughout the simulation time, as evidenced by the movement of the RMSD curve within 2 Å. These plots suggested that each system was quite stable for the docking study. Five equidistant frames for each protein at 20-ns distance (from 100-ns simulation), representing the dynamics of the protein structure, were extracted. Docking studies were then performed on these frames.

### Screening of potential compounds from the US Food and Drug Administration–approved library

The binding affinity of US FDA-approved drugs with each of the proteins was assessed through molecular docking. The average glide docking score (average of five frames) was used to identify potential binders of the individual proteins. The top 20 compounds for each protein, based on their glide score, are given in **Supplementary Table S8**. Details for individual proteins are given below.

**Figure 7:**
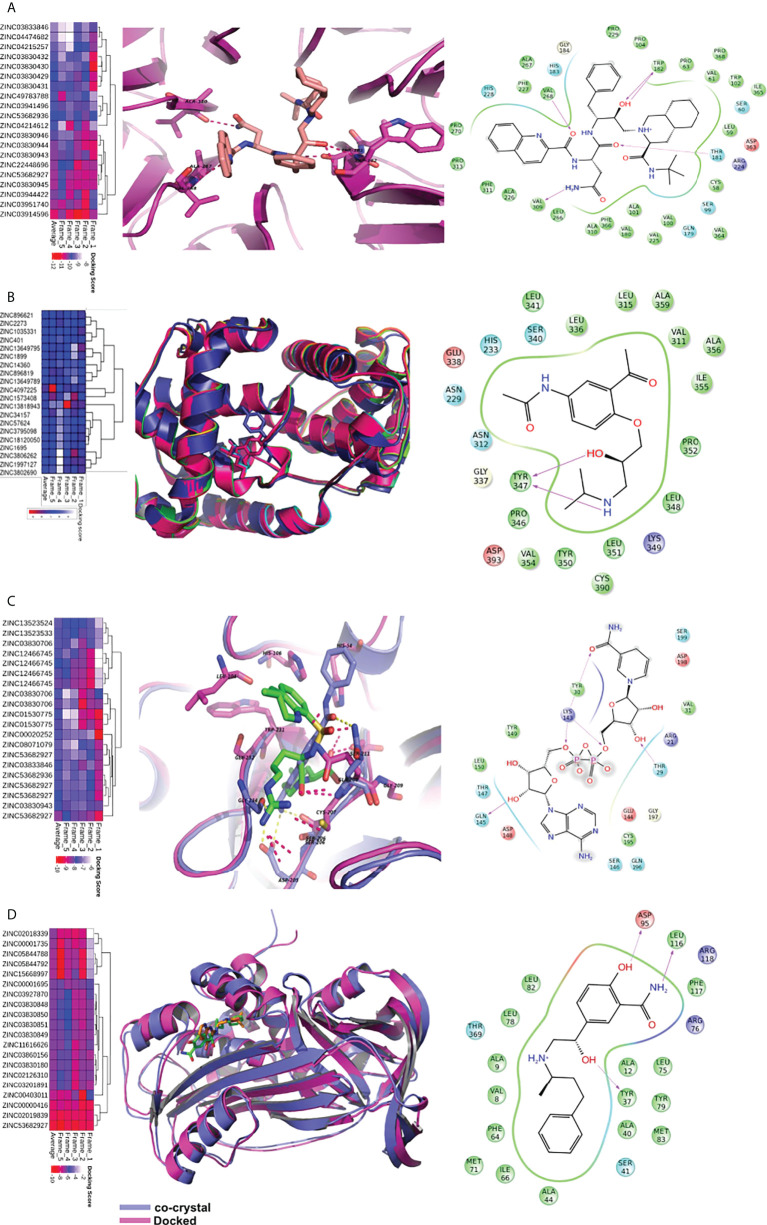
Virtual screening of FDA approved drugs against identified proteins.** **Top scoring 20 molecules for each of the five proteins are shown in heatmap (figures **A–D**). The first column depicts the heatmap of docking scores in five MD frames. The average docking score is also depicted for better understanding. In the second column, proteins are shown in cartoon while the ligands are shown in sticks. The dotted lines depict hydrogen bonds. The third column depicts the interaction map of the ligand receptor interactions. The pink arrows show hydrogen bonds. The arrowhead depicts the HB-acceptor molecule. Pi-pi stacking interactions are depicted by green line. **(A)** The docking of saquinavir with BOP1. It makes hydrogen bonding interactions with backbone of TRP-182, VAL-268, VAL-309, and sidechain of THR-181. **(B)** The binding of molecules in CCNA2. The diacetolol is shown in blue sticks. The ligand makes hydrogen bonds with Tyr347 **(C)** The binding of ligands in PLAU. NADH is shown in binding site of PLAU. It makes hydrogen bonding interactions with THR30, TYR31, LYS154, and SER157. **(D)** The binding of ligands in SERPINE1, and docked molecule labetalol. The drug forms hydrogen bonds with sidechain of ASP-95, TYR37.

BOP1 **is a** RNA-binding protein involved in ribosome biogenesis, cell cycle, and cell proliferation (25). The docking analysis indicated saquinavir to be the best binder with an average docking score of -10.9 **(** **Figure 7A** **)**. The active site of BOP1 is surrounded by multiple beta sheets forming a barrel-like structure. The molecule saquinavir is ensconced in a pocket lined by Trp182, Pro104, Pro229, Pro63, Thr181, Pro368, Gly184, Leu266, Val268, and Val309. Analysis of docking poses indicates that saquinavir has several hydrogen-bonding interactions with residues, *viz.*, Trp182, Val268, and Val309.

CCNA2 (cyclin A2) binds with both CDK2 and CDK1. It is required for entry into the S and M phases of the cell cycle. The overexpression of CCNA2 leads to cell growth and proliferation. Diacetolol was found to be the best binder, with an average docking score of -7.6. It is a beta-blocker used as an antihypertensive and antiarrhythmic agent. The docking study indicated that the ligand diacetolol binds in a cavity lined by residues His233, Gln337, Ser340, Tyr347, Tyr350, Pro352, Val354, Ile355, Ala356, Cys390, Asp393, and Leu394 of CCNA2. It makes HB interaction with Tyr347 (**Figure 7B**). It can be an attractive chemotherapeutic option for OSCC.

*PLAU* encodes a serine protease (uPA) that converts plasminogen to plasmin. uPA is involved in the degradation of the basement membrane and extracellular matrix (26). *PLAU* is one of the potential biomarkers for HNSCC and several other cancers. Nicotinamide adenine dinucleotide hydrogen (NADH), argatroban, diminazene (DIZE), and pentamidine are among the molecules that showed good binding affinity with PLAU. NADH binding to PLAU is shown in **Figure 7C**. It makes hydrogen-bonding interactions with Thr30, Tyr31, Lys154, and Ser157. NADH, due to its role in energy production, may help against wastage and weakness of cancer patients. It is also used to improve mental alertness. It can be given orally and has cleared clinical trials as a chemotherapeutic agent for other illnesses (27).

*SERPINE1* (PAI-I) inhibits the plasminogen activator uPA/uPAR complex that promotes cell matrix degradation and cell migration. Overexpression of *SERPINE1* is highly associated with poor survival in primary tumor, lymph node, and head and neck cancer metastasis. The docking study showed that NADH, reproterol, and labetalol bind to *SERPINE1* with high affinity. The molecule labetalol makes hydrogen-bonding interactions with Tyr37, Ser41, Asp95, Phe117, and Arg118. The phenyl ring sits in the vicinity of hydrophobic residues such as Phe64, Ile66, and Phe117 **(** **Figure 7D** **)**. We could not find a good binder for CKS2. The overall analysis indicates that the identified small molecules hold potential as possible therapeutics for OSCC and other cancers.

### Stability analysis of the drug–ligand complex

Post docking MD simulation was run to check the stability of the ligands inside the binding cavity of the proteins. The stability of the simulation was analyzed by RMSD. The hydrogen bond interactions between ligand and protein were also calculated to check their strength.

**Figure 8:**
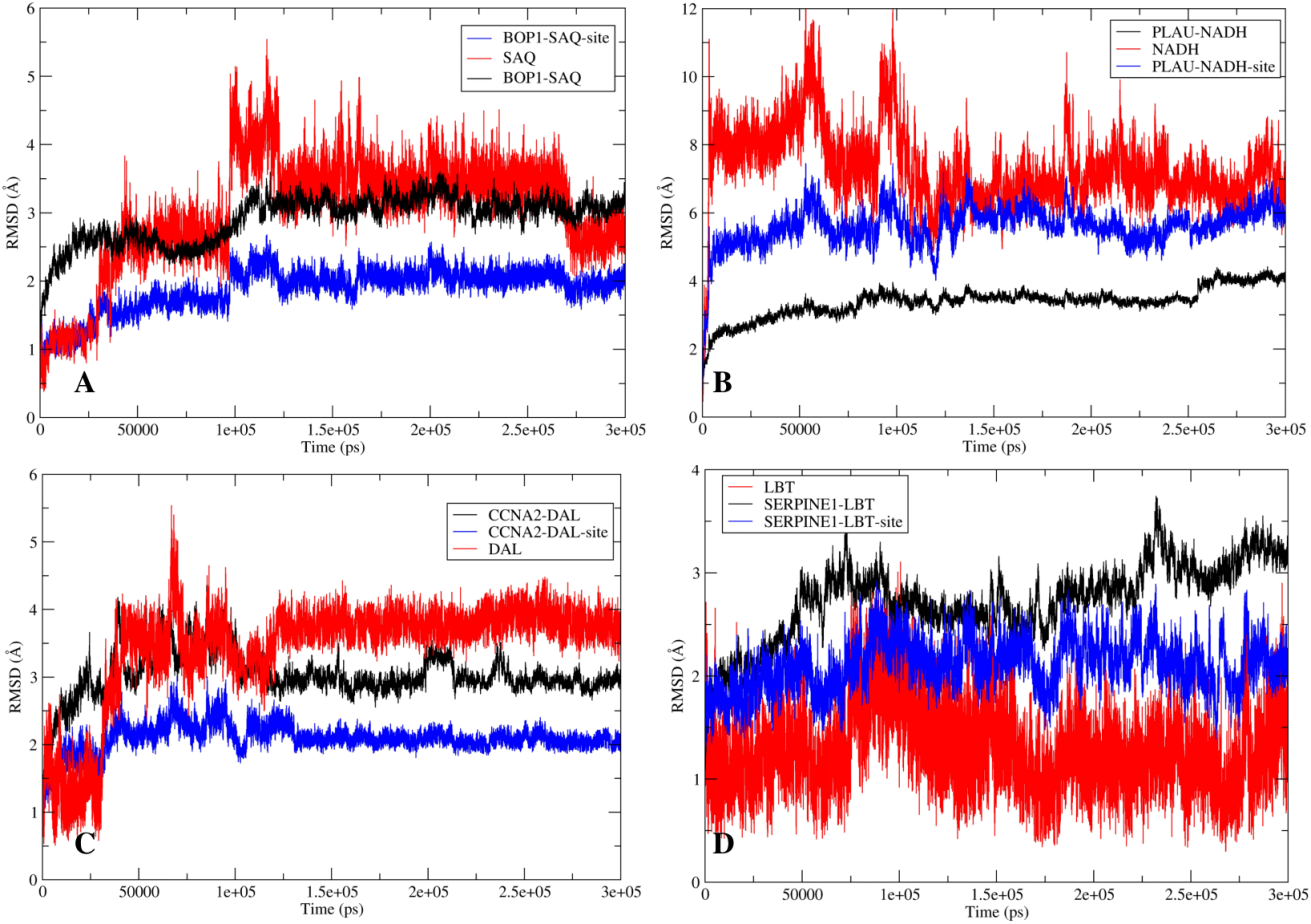
The stability analysis: The root mean square deviation (RMSD) analysis for the protein-ligand complexes. The RMSD of protein is shown in red while the RMSD of ligand is shown in blue. **(A)** RMSD for BOP1-Saquinavir complex. **(B)** RMSD for PLAU-NADH complex. **(C)** RMSD for CCNA2-Diacetolol complex. **(D)** RMSD for SERPINE1-Labetalol complex.

The RMSD analysis of the MD simulation of the BOP1–saquinavir complex indicated that the simulation stabilized at about 10 ns. At about 40 ns, there is a slight change in ligand RMSD (<1 Å). Thereafter, the trajectory is very smooth, and both the protein and ligand RMSDs show a very stable trajectory (**Figure 8A**). The hydrogen bond analysis showed that saquinavir has two hydrogen-bonding interactions (with Thr149 and Trp150) with >50% occupancy (**Table 1**), which indicates that the HB interactions are strong and the BOP1–ligand complex is highly stable.

**Table 1:** Hydrogen bond analysis between the ligand and the protein.

Protein	S. No.	Residue	Ligand	Occupancy*
BOP				
	**1**	THR149	Saquinavir	63.43%
	**2**	TRP150	Saquinavir	63.51%
	**3**	PHE195	Saquinavir	31.31%
**PLAU**				
	**1**	LYS154	NADH	71.71%
	**2**	TYR31	NADH	22.49%
	**3**	SER157	NADH	16.89%
**CCNA2**				
	**1**	TYR176	Diacetolol	19.89%
	**2**	LYS175	Diacetolol	14.47%
**SERPINE1**				
	**1**	ASP95	Labetalol	60.24%
	**2**	TYR37	Labetalol	16.19%

*Defined as the HB interaction present in x% of frames out of a total of 100,000 frames.

The RMSD during the MD simulation of the PLAU–NADH complex initially increases until about 10 ns; afterward, its movement is confined in a small window (<2 Å) (**Figure 8B**). This clearly indicated that the simulation is stable. The ligand RMSD was also calculated. It shows a close trend as that of the protein, again indicating good stability of the ligand inside the binding cavity. Hydrogen bonds of the ligand with Lys154 and Tyr31 showed occupancy of about 71% and 23%, respectively (**Table 1**). All of these observations point to the high stability of the NADH–PLAU complex. The RMSD analysis of the MD simulation of the CCNA2–diacetolol complex indicated that the simulation stabilized quickly, as indicated by the movement of the RMSD in a narrow window. Both the protein and ligand RMSDs show a stable trajectory; however, we could not find a strong hydrogen bonding interaction between diacetolol and CCNA2 (**Figure 8C** and **Table 1**).

The MD simulation of Serpine1 indicated that after an initial increase, the RMSD gets stabilized quickly, as indicated by a rangebound (<2 Å) movement of the RMSD curve (**Figure 8D**). The ligand RMSD was also calculated, and it showed movement in a very narrow window, indicating the stability of the simulation and that of the complex. The HB analysis indicated that the hydrogen bond between the ligand and Asp95 is highly stable, as measured by an occupancy of >60% (**Table 1**). Overall analysis indicates that the Serpine1–labetalol complex is highly stable.

## Discussion

In recent years, prognosis-based gene signature identification has been of immense interest for the prediction of outcome or for evaluation of the course of a disease (28, 29). Therapeutic biomarker prediction models are currently in focus to identify predictive factors of the response to chemotherapy (30, 31). Non-responding cancer cells are either refractory to chemotherapy or have acquired resistance during the course of the treatment. Both are strongly related to molecular alteration of the targets. The major challenge is acquired chemoresistance during treatment, which eventually leads to cancer regrowth, even if the tumor initially responds to the chemotherapeutic agent. Moreover, reports suggest changes in gene and protein expression levels in cancer tissues and cell lines after chemotherapy. For instance, a xenograft study has shown that drug treatment (5-fluorouracil and cisplatin) first reduces the expression level of drug-specific sensitivity-related genes followed by their upregulation in the regrowth phase and emergence of chemoresistance in esophageal cancer (32). Chemoresistance, either intrinsic or acquired, contributes to the low survival of the patients, necessitating the need for identification of drug-specific sensitivity-related markers and their modulators to enhance the sensitivity of cancer cells to subdue/delay chemoresistance. One such example is inhibition of the Phosphoinositode-3-kinase/AKT serine/threonine kinase (PI3K/AKT) pathway to increase the sensitivity and reverse acquired resistance of esophageal cancer cells to chemotherapeutic drugs (33). Thus, it is imperative to identify the therapeutic targets that not only are advantageous in predicting the clinical outcome but also can resolve the emerging chemotherapy-related issues as well.

In our study, using different genomic data, we examined the changes in gene expression before and after treatment to investigate the key genes, pathways, biomarkers, and risk gene signature. Interestingly, we found that most of the genes that were highly expressed in OSCC patients are found to be significantly downregulated after doxorubicin treatment. We also get another cluster of genes whose expression does not change significantly after doxorubicin treatment. In fact, the expression of some of them increases further. In total, 168 overlapping DEGs were found from the three datasets and were considered for further study. Five gene signatures (*SERPINE1*, *PLAU*, *BOP1*, *CKS2*, and *CCNA2*) were proposed in this study through Cox regression and machine learning. The overexpression of these genes increases the risk of adverse outcome. Application of Cox regression resulted in an equation that can be used for risk stratification of OSCC patients. From the survival analysis, we were able to predict that increased expression of these genes is related to poor prognosis of the patients. A similar study found SMA and SERPINE1 to be significantly associated with prognosis in OSCC (34); SERPINE1, PLAU, and ACTA1 acted as both diagnostic and prognostic markers (35). Another study by Liu et al. (36) identified an eight-gene prognostic signature in HNSCC that included PLAU. CCNA2 is identified as an independent indicator of worse OS and may serve as a reliable biomarker to identify high-risk subgroups with poor prognosis in OSCC (37). Moreover, these five genes were highly expressed and reported to be associated with cancer progression in various cancers including HNSCC (35, 38), glioblastoma multiforme (GBM) (39), epithelial ovarian cancer (40, 41), gastric cancer (42, 43), breast cancer (44, 45), bladder cancer (46, 47), esophageal cancer (48), colorectal cancer (49, 50), hepatocellular carcinoma (51), melanoma (52), and non-small cell lung carcinoma (NSCLC) (53). In cancer pathogenesis, these genes perturbed numerous cellular mechanisms such as extracellular matrix (ECM) modulation, epithelial-to-mesenchymal transition (EMT), cell migration, and angiogenesis (39, 47–49, 54–59). Interestingly, from a genetic study, we also found that gene amplification in *SERPINE1*, *PLAU*, *BOP1*, and *CKS2* is the primary cause of their overexpression, except in *CCNA2*, where mutation is the predominant cause in HNSCC. Experimental evidence showed that amplification of these genes occurs at both mRNA and protein levels in several cancers such as breast cancer (60, 61), prostate cancer (62), rectal cancer (63), hepatocellular cancer (51), NSCLC (64), gastric cancer (65), and tongue cancer (66).

**Figure 9:**
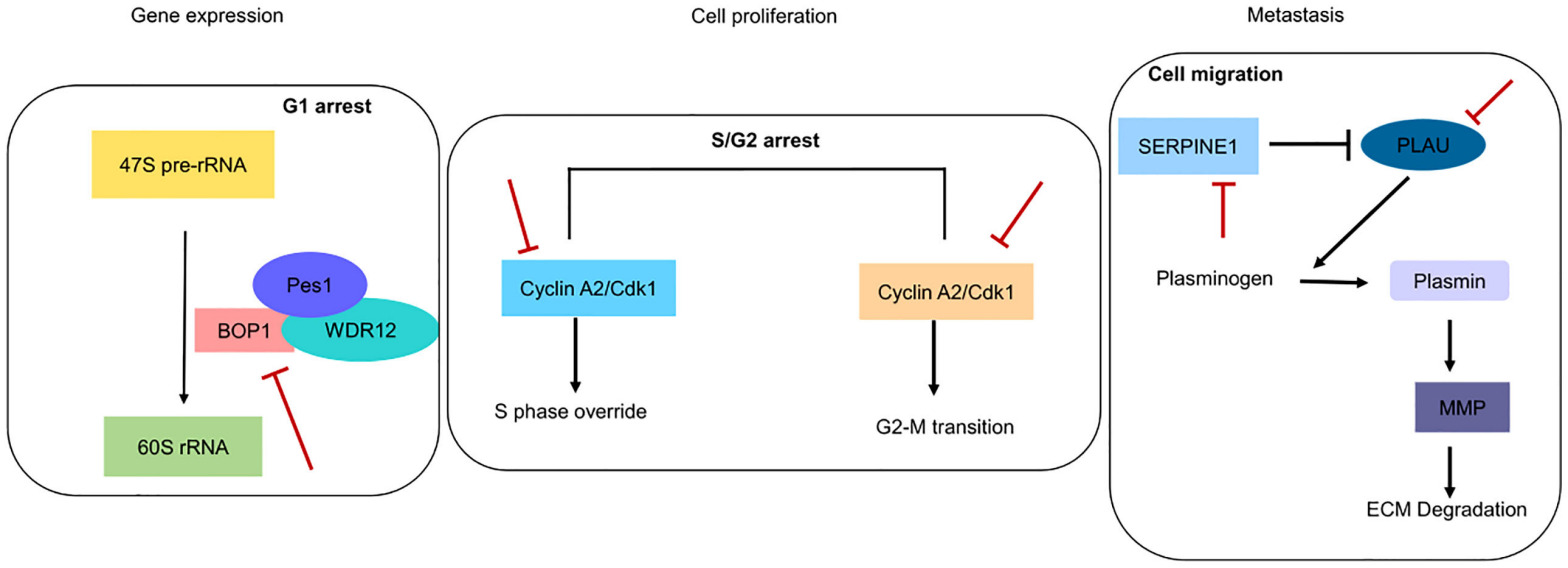
Pictorial depiction of identified genes in biological pathways. Red blunt lines indicate that their pharmacological inhibition can inhibit major functions involved in gene expression, cell proliferation and metastasis.

Overexpression of these markers is indicative of poor prognosis and corresponds to chemotherapy resistance. We showed that initially during chemotherapy, there is downregulation of these genes in the responsive cancer (**Figure 5C**), but as treatment continues, these genes are upregulated to give rise to chemoresistant phenotypes (**Figure 5D**). We examined the mRNA expression of these five genes with different drug treatments, which include doxorubicin, cisplatin, oxaliplatin, gemcitabine, topotecan, temozolomide, paclitaxel, JQ1, romidepsin, dauricine, and SNSO32, in various cancers to support our outcome. All of the drugs that we used in our study have DNA as their target and inhibit DNA replication or transcription. In another analysis with the drug olaparib, which is a poly(ADP-ribose) polymerase 1 (PARP1) inhibitor (GSE165585), we found that our genes of interest were not significantly expressed (**Supplementary Table S3**). In support of our results, we found that PARP1 inhibitors help in the sensitization of temozolomide-resistant glioblastoma cancer (67). We found that after drug treatment, gene expression changes from high to low or low to high or remains unchanged. Further additional new gene expression was also seen. We found that during the period of treatment responsiveness, our genes change their expression level from low to high again, which is the same as the prior treatment. Other studies have also determined the involvement of these genes in resistance generation, for example, *SERPINE1* is upregulated in cisplatin-resistant oral cancer cell lines (SCC9, SCC4, and H357) (68) and paclitaxel-resistant breast cancer (44). In the context of therapeutic potential, SERPINE1 has been identified as a potential therapeutic target, as it acts as a pro-proliferative oncogenic factor (69). *PLAU* is found to be upregulated in cisplatin-resistant oral cancer cell lines (68). Both *PLAU* and *SERPINE1* were found to be highly expressed in breast cancer patients with adjuvant endocrine therapy and related to shorter disease-free survival and OS (70). Whereas *in vivo*, BOP1 downregulation was reported to inhibit paclitaxel resistance and Cancer stem cells (CSC)-like phenotype in triple-negative breast cancer (TNBC) cells (71). In estrogen receptor–positive (ER+) breast cancer, tamoxifen resistance is correlated with the overexpression of *CCNA2* (72). CKS2 in complex with SSPB1 regulates mitochondrion DNA replication in cervical cancer and can be indicative of chemoradioresistance (73). In the present study, we found that these genes are upregulated and are related to resistance, and they are involved in vital biological processes (**Figure 9**); therefore, targeting these genes can be of immense therapeutic benefit.

Thus, it is imperative to identify modulators that can check aberrant expression of these genes and increase the barrier toward the emergence of chemoresistance. In this milieu, we also identified small-molecule ligands that can enhance the efficacy/sensitivity of chemotherapeutic agents. A combination of molecules targeting proteins can provide a potent therapeutic option with reduced changes of emergence of chemoresistance. Many of the identified molecules are already reported to be effective against cancers such as colorectal, breast, and lung. Some of the selected proteins are involved in mutually exclusive pathways, as evident from the network analysis, and thus have different mechanisms of action. Simultaneously targeting them can be advantageous to both primary tumor and advanced metastatic tumors. Most of the top-scoring molecules for BOP1 were HIV protease inhibitors, e.g., saquinavir, indinavir, nelfinavir, and ritonavir (Norvir). They are reported to induce cell death in both the chemosensitive and chemoresistant ovarian cancer cell lines in a dose-dependent manner (74). The lopinavir/ritonavir combination is reported to have significant inhibition on cell growth and migration, whereas it enhanced radiosensitivity in HNSCC cell lines (75). The anticancer potential of protease inhibitors is already reported in various previous publications. Some of these molecules are in clinical trials. Therefore, they may present attractive options as targeted chemotherapeutic agents against chemosensitive and chemoresistant OSCC as well (76–78). Moreover, BOP1 inhibition provides additional advantage for non-cancerous cells by inducing a cytoprotective nucleolar stress response and reducing damage to normal tissues from anticancer drugs such as camptothecin or methotrexate. It is reported that *BOP1* inhibition together with camptothecin results in selective killing of p53 null cells, producing a synergistic effect (79, 80). *PLAU* encodes uPA, commonly associated with cancer progression *via* apoptosis inhibition and breakdown of the ECM. It also promotes angiogenesis (81). PLAU, as a top identified molecule, inhibits various biological functions such as NADH due to its role in energy production and may help against wastage and weakness of cancer patients. It is also used to improve mental alertness. It can be given orally and has cleared clinical trials as a chemotherapeutic agent for other illnesses (27). Argatroban, which is an antithrombotic agent, inhibits the metastasis in breast cancer (82) and melanomas (83). It has significant antineoplastic effect on gliomas as well (84). It downregulates the MAPK/ERK and STAT phosphorylation that results in a reduction of interleukin (IL)-6, IL-12, and tumor necrosis factor (85). The antiparasitic pentamidine has been shown to be effective against various human cancers such as melanoma (86), breast cancer (87), lung cancer, ovarian cancer, and cervical cancer (88, 89). However, the mechanism of its antineoplastic action has not been elucidated fully. Overall, these results infuse great confidence in our analysis. Labetalol, which we have identified as a SERPINE1 binder, blocks both alpha and beta adrenoceptors and has been used for the treatment of hypertension. Alpha-blockers have been reported to increase recurrence-free survival (RFS) (90). Recently, it has been suggested that beta-blockers hinder mechanisms that initiate tumorigenesis, angiogenesis, and metastasis. Beta-blockers have shown good antineoplastic activity in various cancer cell lines. They are also reported to increase the effect of anticancer chemotherapy (91, 92). Therefore, labetalol, having a mix of alpha- and beta-blocker activities, can be a potential candidate for the treatment of OSCC. Reproterol is a β2-agonist used as an antiasthmatic drug. Thus, we suggest that targeting BOP1 and/or PLAU can be advantageous against both primary and metastatic tumors. Their inhibitors can also be combined together with fluorouracil and methotrexate (93). We have also identified CCNA2 ligands, whereas no direct small-molecule inhibitors for CCNA2 are yet known. While targeting the CCNA2 function, we will be able to target the S/G2/G2-M phase of the cell cycle.

## Conclusion

In this study, we have identified changes in the gene expression level as a result of treatment in OSCC. Applying machine learning techniques and Cox regression, we constructed a five-gene-based prognostic signature that can stratify the patients (high and low risk). It is evident that overexpression of these genes is related to poor prognosis and reduced survival in many cancers including OSCC. They are also related to the emergence of chemoresistance against many drugs. Changes in their expression levels, pretreatment, posttreatment, and resistant cell lines make them suitable for targeting. We have also identified potential molecules that can bind to these proteins with high affinity. Since the identified proteins are involved in disparate processes, a combination of molecules targeting them can provide a potent therapeutic option with reduced chances of chemoresistance. We hope that this study provides new avenues for the design of better chemotherapeutic agents especially against chemoresistance in OSCC.

## Data availability statement

The datasets presented in this study can be found in online repositories. The names of the repository/repositories and accession number(s) can be found in the article/**Supplementary Material**.

## Author contributions

The work was designed and conceptualized by PK and AD. PK, SK, AD, SB, MS, and BM analyzed the data and performed the work. The manuscript was prepared and written by PK, SK, and AD. All authors read and approved the final manuscript.

## Acknowledgments

The authors acknowledge the Director, Institute of Life Sciences (ILS), Bhubaneswar and the Department of Biotechnology, Government of India for providing necessary high-performance computing facility. PK and SK are thankful for the University Grant Commission (UGC) for their fellowship. SB and BM is thankful for CSIR for their fellowship. The ILS high performance computing facility is duly acknowledged for providing computational facility for this work. This work is supported by an ILS core grant.

## Conflict of interest

The authors declare that the research was conducted in the absence of any commercial or financial relationships that could be construed as a potential conflict of interest.

## Publisher’s note

All claims expressed in this article are solely those of the authors and do not necessarily represent those of their affiliated organizations, or those of the publisher, the editors and the reviewers. Any product that may be evaluated in this article, or claim that may be made by its manufacturer, is not guaranteed or endorsed by the publisher.

## Abbreviations

ACC, adrenocortical carcinoma; BLCA, bladder urothelial carcinoma; BRCA, breast invasive carcinoma; CESC, cervical squamous cell carcinoma; COAD, colon adenocarcinoma; DLBC, diffuse large B-cell lymphoma; ESCA, esophageal carcinoma; GBM, glioblastoma multiforme; HNSCC, head and neck squamous cell carcinoma; KICH, kidney chromophobe; KIRC, kidney renal clear cell carcinoma; KIRP, kidney renal papillary cell carcinoma; AML, acute myeloid leukemia; LGG, brain lower-grade glioma; LIHC, liver hepatocellular carcinoma; LUAD, lung adenocarcinoma; LUSC, lung squamous cell carcinoma; OV, ovarian serous cystadenocarcinoma; PAAD, pancreatic adenocarcinoma; READ, rectum adenocarcinoma; SARC, sarcoma; SKCM, skin cutaneous melanoma; STAD, stomach adenocarcinoma; TGCT, testicular germ cell tumor; THCA, thyroid carcinoma; THYM, thymoma; UCEC, uterine corpus endometrial carcinoma; UCS, uterine carcinosarcoma; MESO, mesothelioma; UVM, uveal melanoma; GEM, gemcitabine; GC, gastric cancer; OC, ovarian cancer, TMZ, temozolomide; NSCLC, non-small cell lung carcinoma.
