# Supplementary material for: Identification of therapeutically potential targets and their ligands for the treatment of OSCC
Source: Front Oncol. 2022 Sep 20;12:910494. doi: 10.3389/fonc.2022.910494 (PMC9530560; doi:10.3389/fonc.2022.910494)
Supplement: Supplementary file 1 [file DataSheet_1.docx]

**
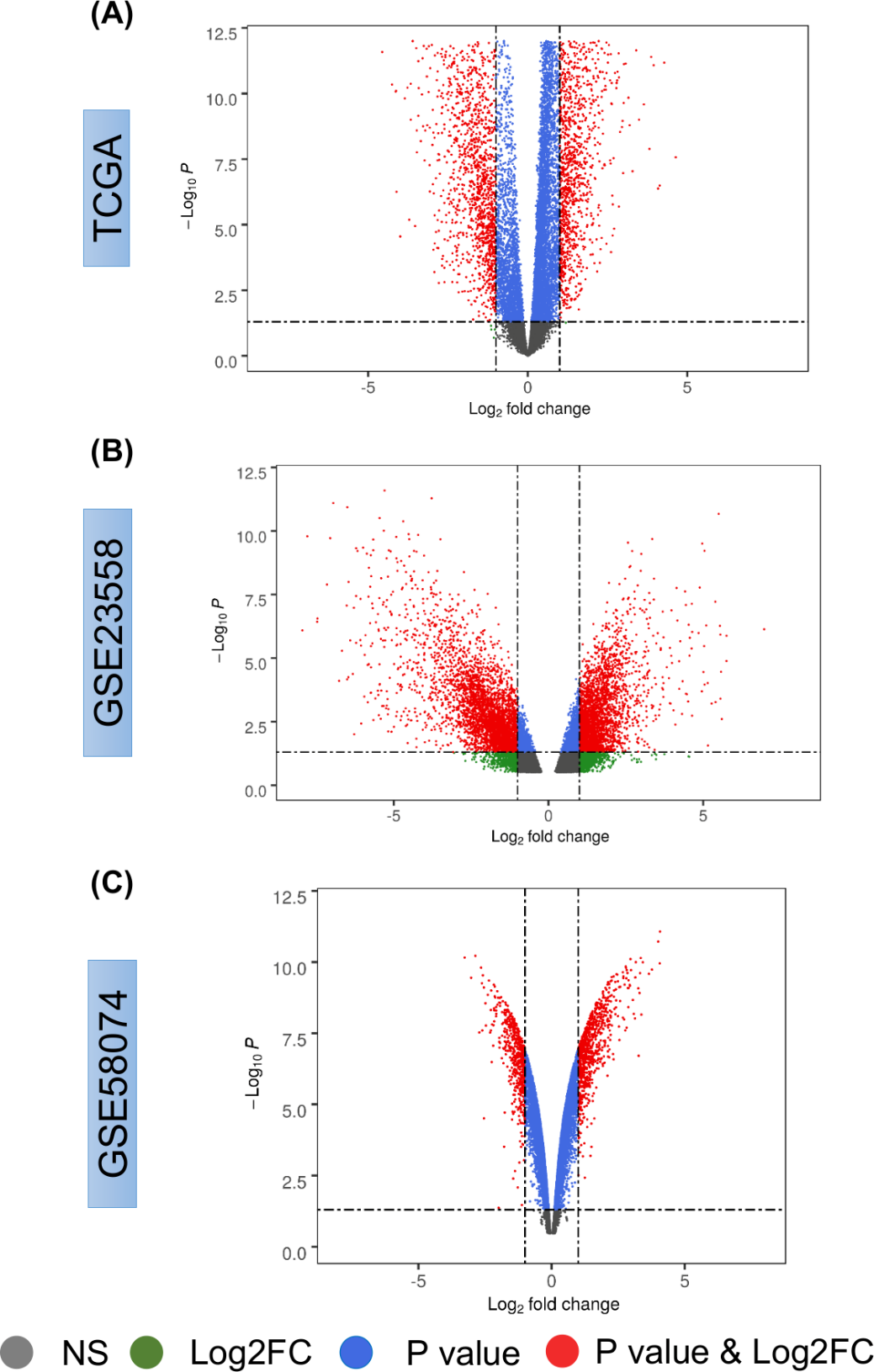
Supplementary Figures**

**Supplementary figure 1:** Volcano Plot depicting differentially expressed genes from TCGA, GSE23558, and GSE58074. DEGs were considered signification based on *p*-value < 0.05 and log2 (fold-change) ≥ 1. Colored dots correspond to individual genes whose expression differences were significant based on both p and logFC value (red), only *p*-value (blue), only logFC (green), or not significant (grey).


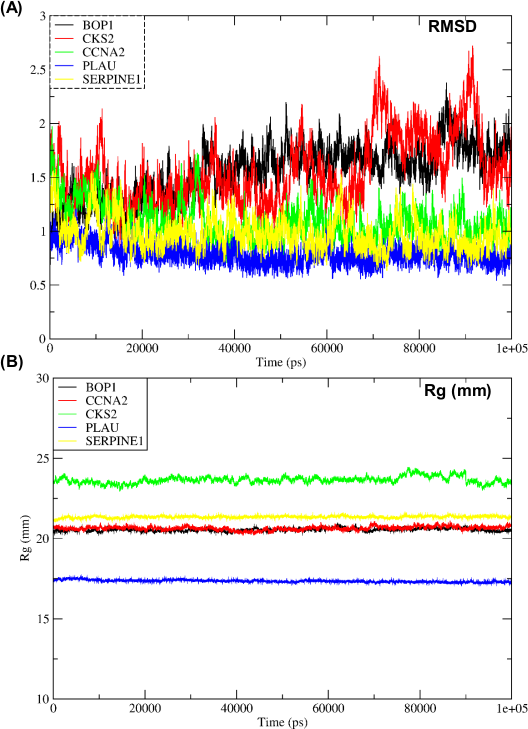


**Supplementary figure 2:** Characterization of the GO terms and pathways associated with identified common genes. Percentage of Go terms and pathways related to common functions are represented.


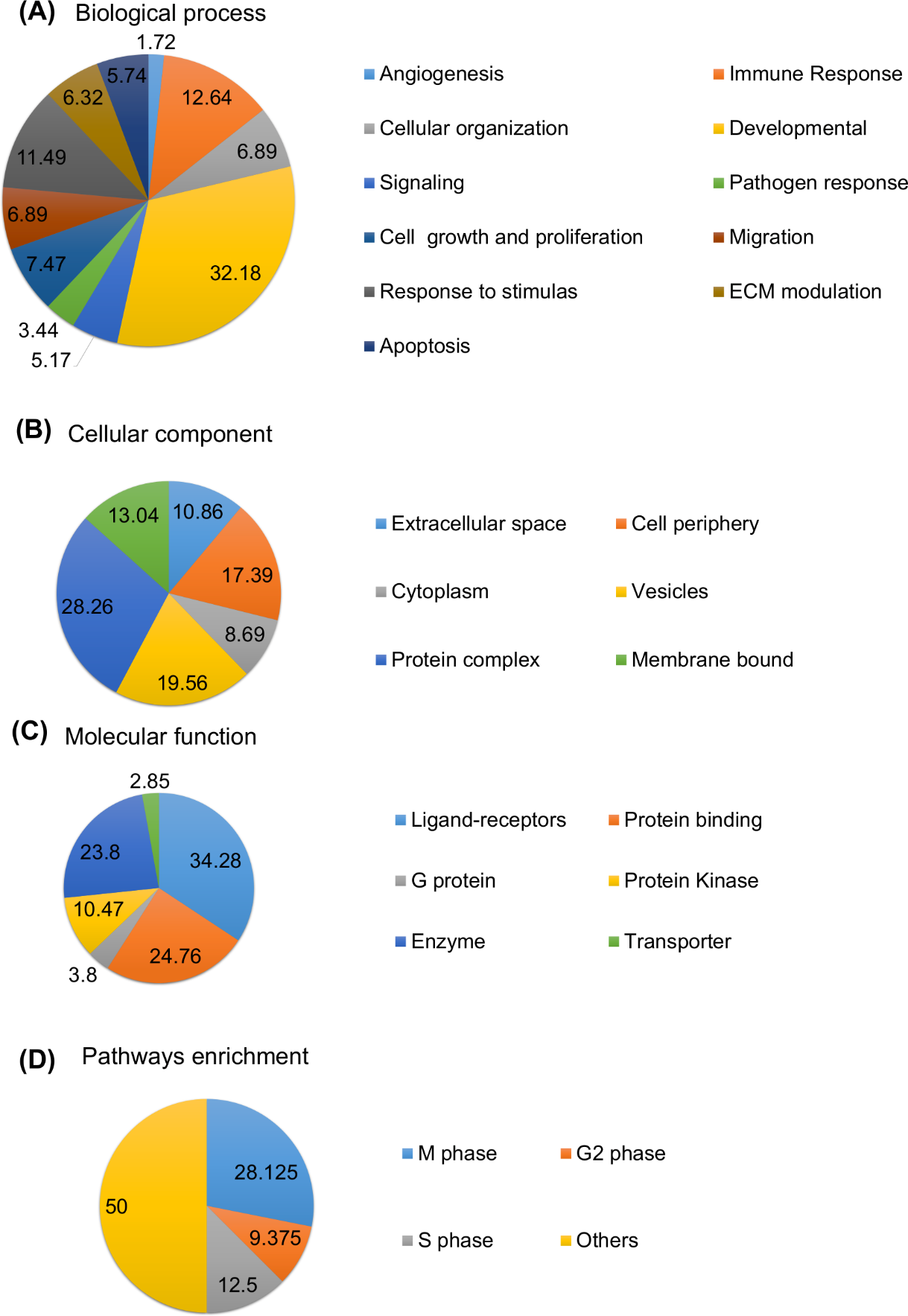

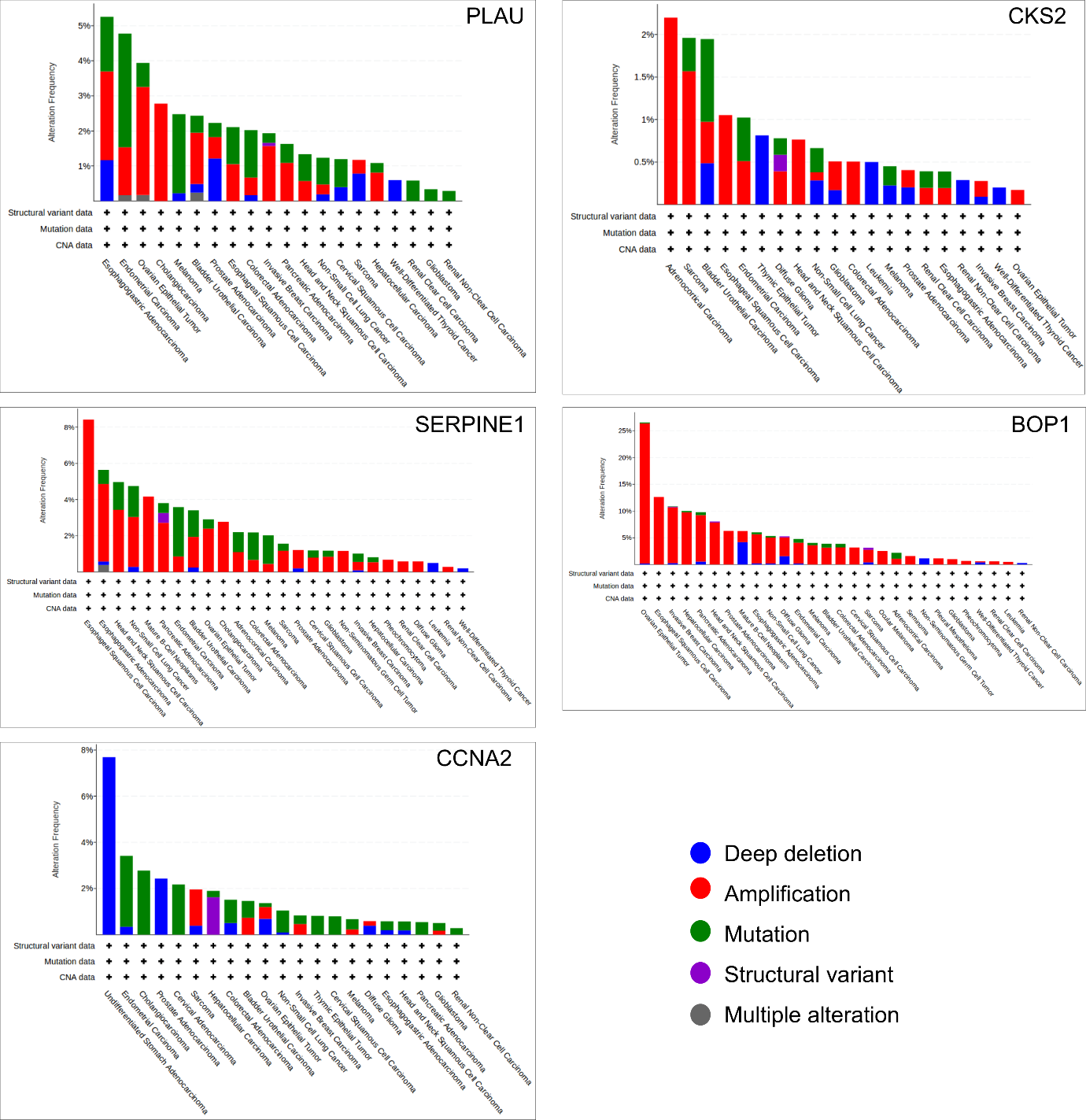


**Supplementary figure 3:** Analysis of genetic alterations using cBioPortal data. The figure was plotted using cBioPortal website and depicts the different types of genetic alteration present in PLAU, CKS2, SERPINE1, BOP1 and CCNA2 across various cancers.
