# Supplementary material for: Identification of therapeutically potential targets and their ligands for the treatment of OSCC
Source: Front Oncol. 2022 Sep 20;12:910494. doi: 10.3389/fonc.2022.910494 (PMC9530560; doi:10.3389/fonc.2022.910494)
Supplement: Supplementary file 2 [file Table_1.docx]

**Supplementary table 1 :** International classification of diseases 10 (ICD 10) code used by TCGA. Oral cancer sample were selected based on these codes.

| **ICD 10 code** | **Sites** |
| --- | --- |
| C00.9 | Lip |
| C01 | Base of tongue |
| C02.1 | Border of tongue |
| C02.9 | Tongue |
| C03.0 | Upper gum |
| C03.1 | Lower gum |
| C03.9 | Gum |
| C04.0 | Anterior floor of mouth |
| C04.9 | Floor of mouth |
| C05.0 | Hard Palate |
| C05.9 | Palate |
| C06.0 | Cheek mucosa |
| C06.9 | Mouth |
| C14.8 | Over lapping lesions of lip & oral cavity |
