# Supplementary material for: Identification of therapeutically potential targets and their ligands for the treatment of OSCC
Source: Front Oncol. 2022 Sep 20;12:910494. doi: 10.3389/fonc.2022.910494 (PMC9530560; doi:10.3389/fonc.2022.910494)
Supplement: Supplementary file 3 [file Table_2.docx]

| **TCGA sample information** | |
| --- | --- |
| No. of OSCC samples | Tumor = 319  NAT = 44 |
| Sex | Female = 105  Male = 214 |
| Status | Alive = 169  Dead = 150 |
| Stage | I = 21  II = 81  III = 68  IV = 158 |
| Tumor size (T) | T1 = 21  T2 = 108  T3 = 84  T4 = 105 |
| Node (N) | N0 = 161  N1 = 59  N2 = 90  N3 = 5 |
| Metastasis (M) | M0 = 310  M1 = 2 |
| HPV status | Negative = 266  Positive = 43 |

**Supplementary table 2:** Patient’s information with associated clinicopathological features.
