# Supplementary material for: Identification of therapeutically potential targets and their ligands for the treatment of OSCC
Source: Front Oncol. 2022 Sep 20;12:910494. doi: 10.3389/fonc.2022.910494 (PMC9530560; doi:10.3389/fonc.2022.910494)
Supplement: Supplementary file 6 [file Table_5.docx]

**Supplementary table 5:** Robustness of prediction of top 10 predictors identified using RF and PLS.

| **Random Forest (RF)** | **Robustness** | **Partial Least Square Regression (PLS)** | **Robustness** |
| --- | --- | --- | --- |
| SERPINE1 | 100 | PDPN | 100 |
| CCNA2 | 98.04 | CCM2 | 98.3 |
| RND1 | 91.63 | NTF3 | 96.55 |
| BNC1 | 86 | CKS2 | 92.42 |
| PLAU | 85.84 | TNFRSF12A | 85.82 |
| NCS1 | 85.24 | CCNA2 | 85.49 |
| IFI6 | 84.93 | PGD | 80.63 |
| RTKN | 83.31 | PLAU | 73.75 |
| BOP1 | 81.76 | CRTAC1 | 71.55 |
| TENM3 | 79.94 | CYP1B1 | 70.35 |
