# Supplementary material for: Identification of therapeutically potential targets and their ligands for the treatment of OSCC
Source: Front Oncol. 2022 Sep 20;12:910494. doi: 10.3389/fonc.2022.910494 (PMC9530560; doi:10.3389/fonc.2022.910494)
Supplement: Supplementary file 7 [file Table_6.docx]

| **Cancer** | **SERPINE1** | **PLAU** | **CKS2** | **CCNA2** | **BOP1** |
| --- | --- | --- | --- | --- | --- |
| ACC | NA | NA | 1.643 | 1.197 | NA |
| BLCA | NA | 1.729 | 2.594 | 2.582 | 1.096 |
| BRCA | NA | 1.124 | 3.053 | 2.097 | NA |
| CESC | NA | 3.098 | 4.298 | 2.713 | NA |
| COAD | NA | 1.991 | 3.534 | 2.574 | 1.427 |
| DLBC | 2.635 | 3.666 | 5.289 | 3.918 | 3.787 |
| ESCA | 3.371 | 2.265 | 1.564 | 1.429 | NA |
| GBM | 4.993 | 3.801 | 3.701 | 2.524 | NA |
| HNSC | 3.651 | 3.232 | 1.45 | 1.208 | 1.285 |
| KICH | -2.575 | -3.363 | 1.293 | NA | NA |
| KIRC | 1.792 | -1.297 | NA | 1.159 | NA |
| KIRP | -1.678 | NA | NA | NA | NA |
| LAML | -1.484 | NA | -1.985 | -2.672 | NA |
| LGG | 1.608 | 1.014 | 1.712 | NA | NA |
| LIHC | -1.115 | 1.11 | 1.75 | 1.445 | 1.532 |
| LUAD | -1.802 | 1.321 | 1.296 | 1.501 | NA |
| LUSC | NA | 2.139 | 2.121 | 2.74 | 1.144 |
| OV | -2.032 | 3.259 | 4.009 | 1.76 | 1.296 |
| PAAD | 3.555 | 4.876 | 4.002 | 2.175 | NA |
| READ | NA | 2.012 | 3.748 | 2.524 | 1.329 |
| SKCM | NA | NA | 2.699 | 1.753 | 1.149 |
| STAD | 1.776 | 2.191 | 3.723 | 3.019 | 1.272 |
| TGCT | NA | 3.57 | NA | 1.64 | 1.06 |
| THCA | -1.001 | 1.855 | 2.072 | NA | -1.813 |
| THYM | 1.209 | 1.214 | 4.858 | 3.724 | 3.006 |
| UCEC | -1.027 | NA | 4.241 | 1.954 | NA |
| UCS | NA | NA | 4.403 | 1.998 | 1.465 |

**Supplementary table 6:** Significant mRNA expression of five genes across different cancers.
