# Supplementary material for: Identification of therapeutically potential targets and their ligands for the treatment of OSCC
Source: Front Oncol. 2022 Sep 20;12:910494. doi: 10.3389/fonc.2022.910494 (PMC9530560; doi:10.3389/fonc.2022.910494)
Supplement: Supplementary file 8 [file Table_7.docx]

**Supplementary table 7:** List of GEO ID taken for DGEs analysis for drug-sensitive and resistant cell lines.

| **Case type** | **GEO ID** | **Drug name** | **Cell line** | **Cancer** |
| --- | --- | --- | --- | --- |
| Sensitive cell line | GSE63529 | SNS032 | Primary culture | High grade serous ovarian cancer |
|  | GSE133120 | Romidepsin | A549 | Lung cancer |
|  | GSE176162 | Dauricine | BxPC3 | Pancreatic cancer |
|  | GSE124597 | Doxorubicin | MCF7 | Breast cancer |
|  | GSE76092 | Oxaliplatin | Ht29 | Colorectal cancer |
|  | GSE66549 | Cisplatin | H1869 | Lung Squamous Cell Carcinoma |
| Resistant cell line | GSE153679 | Temozolomide (TMZ) | 559T | Glioblastoma |
|  | GSE153679 | Temozolomide (TMZ) | 592T | Glioblastoma |
|  | GSE164813 | JQ1 (Thienotriazolodiazepine) | H23 | Lung adenocarcinoma |
|  | GSE58118 | Gemcitabine | MNK28 | Gastric cancer |
|  | GSE58472 | Cisplatin | IGROV1 | Ovarian cancer |
|  | GSE58472 | Oxaliplatin | IGROV1 | Ovarian cancer |
|  | GSE73935 | Paclitaxel | Primary cell line | Ovarian cancer |
|  | GSE73935 | Topotecan | A2780 | Ovarian cancer |
|  | GSE73978 | Cisplatin | AsPc1 | Pancreatic ductal adenocarcinoma |
|  | GSE80617 | Gemcitabine | Panc1 | Pancreatic cancer |
